# Characterization of outbreak response strategies and potential vaccine stockpile needs for the polio endgame

**DOI:** 10.1186/s12879-016-1465-7

**Published:** 2016-03-24

**Authors:** Radboud J. Duintjer Tebbens, Mark A. Pallansch, Steven G. F. Wassilak, Stephen L. Cochi, Kimberly M. Thompson

**Affiliations:** Kid Risk, Inc., 10524 Moss Park Rd., Ste. 204-364, Orlando, FL 32832 USA; Division of Viral Diseases, National Center for Immunization and Respiratory Diseases, Centers for Disease Control and Prevention, Atlanta, GA USA; Global Immunization Division, Center for Global Health, Centers for Disease Control and Prevention, Atlanta, GA USA

**Keywords:** Polio, Eradication, Risk management, Vaccine, Stockpile

## Abstract

**Background:**

Following successful eradication of wild polioviruses and planned globally-coordinated cessation of oral poliovirus vaccine (OPV), national and global health leaders may need to respond to outbreaks from reintroduced live polioviruses, particularly vaccine-derived polioviruses (VDPVs). Preparing outbreak response plans and assessing potential vaccine needs from an emergency stockpile require consideration of the different national risks and conditions as they change with time after OPV cessation.

**Methods:**

We used an integrated global model to consider several key issues related to managing poliovirus risks and outbreak response, including the time interval during which monovalent OPV (mOPV) can be safely used following homotypic OPV cessation; the timing, quality, and quantity of rounds required to stop transmission; vaccine stockpile needs; and the impacts of vaccine choices and surveillance quality. We compare the base case scenario that assumes aggressive outbreak response and sufficient mOPV available from the stockpile for all outbreaks that occur in the model, with various scenarios that change the outbreak response strategies.

**Results:**

Outbreak response after OPV cessation will require careful management, with some circumstances expected to require more and/or higher quality rounds to stop transmission than others. For outbreaks involving serotype 2, using trivalent OPV instead of mOPV2 following cessation of OPV serotype 2 but before cessation of OPV serotypes 1 and 3 would represent a good option if logistically feasible. Using mOPV for outbreak response can start new outbreaks if exported outside the outbreak population into populations with decreasing population immunity to transmission after OPV cessation, but failure to contain outbreaks resulting in exportation of the outbreak poliovirus may represent a greater risk. The possibility of mOPV use generating new long-term poliovirus excretors represents a real concern. Using the base case outbreak response assumptions, we expect over 25 % probability of a shortage of stockpiled filled mOPV vaccine, which could jeopardize the achievement of global polio eradication. For the long term, responding to any poliovirus reintroductions may require a global IPV stockpile. Despite the risks, our model suggests that good risk management and response strategies can successfully control most potential outbreaks after OPV cessation.

**Conclusions:**

Health leaders should carefully consider the numerous outbreak response choices that affect the probability of successfully managing poliovirus risks after OPV cessation.

## Background

As the areas of endemic wild poliovirus (WPV) circulation shrink and the numbers of cases decline globally, focus continues to shift toward the polio endgame [1]. Preparing for oral poliovirus vaccine (OPV) cessation and managing potential outbreaks emerge as critical activities [2]. The Global Polio Eradication Initiative (GPEI) recognizes the need to develop outbreak response plans for the polio endgame as a priority [1], but efforts to date remain primarily qualitative.

Prior analysis of outbreak response strategies for polio demonstrated the importance of rapid detection and response [3]. Another analysis showed the importance of sufficiently high population immunity to transmission prior to OPV cessation to prevent the subsequent formation of circulating vaccine-derived polioviruses (cVDPVs) [4]. This study also demonstrated that responding with monovalent OPV (mOPV) to a cVDPV outbreak that occurs shortly after homotypic OPV cessation in a closed model outbreak community does not lead to continued circulation of the introduced OPV virus if the outbreak response shuts down the outbreak virus [4]. However, outbreak response planning efforts need to consider the potential risks of exporting into other areas either the outbreak virus or the OPV virus used for outbreak response. In this context, the timing of a virus reintroduction after global OPV cessation and the resulting accumulation of individuals who can contribute to transmission in the event of an outbreak will likely affect decisions related to using a live virus (i.e., mOPV) versus inactivated poliovirus vaccine (IPV) for outbreak response. A recent integrated global model (i.e., the global model) explored the health and economic outcomes associated with phased OPV cessation of the three poliovirus serotypes with different policies with respect to IPV use [2]. The global model deterministically characterized OPV evolution and the emergence of cVDPVs and stochastically simulated potential reintroductions from immunodeficiency-associated vaccine-derived polioviruses (iVDPVs) and (un)intentional releases [2]. The model assumed a very aggressive outbreak response strategy involving 4–6 rounds (and more if transmission continued) with serotype-specific mOPV during the first 5 years after homotypic OPV cessation, or IPV more than 5 years after homotypic OPV cessation [2]. The geographical scope of the outbreak response included the outbreak population of approximately 10 million people for settings with a basic reproduction number (R_0_) for serotype 1 WPV (WPV1) of up to 9. For areas with higher inherent poliovirus transmissibility (i.e., R_0_ for WPV1 > 9), the outbreak response area included the approximately 10 million people in the outbreak population and 9 connected populations each with approximately 10 million people. For all outbreak response efforts, the targeted age groups increased as a function of time since homotypic OPV cessation [2]. With a response delay of 45 days after the initial outbreak detection, the outbreak response strategy successfully stopped all outbreaks in 98 out of a set of 100 stochastic iterations (i.e., realizations of random poliovirus reintroductions and exportations between populations) for a policy involving at least one IPV routine immunization (RI) dose for at least 5 years after cessation of all regular OPV use (i.e., policy abbreviation IPV5) [2]. A related analysis of vaccine needs demonstrated the linkage between pre-OPV cessation vaccine usage and expected vaccine needs from the stockpile to respond to cVDPV outbreaks [5].

The GPEI developed a qualitative matrix of outbreak response plans based on the endgame phase (i.e., <2 years, 3–5 years, and >5 years since coordinated OPV cessation) and area where the outbreak occurs [6]. Specifically, the GPEI recognized areas with a clear history of sustained WPV transmission or the development of cVDPV outbreaks as high-risk, areas with consistently low immunization coverage and/or demonstrated compromised population immunity to disease based on a history of importation of WPV as medium-risk, and areas with consistently higher coverage and few risk factors for sustained transmission of poliovirus as relatively low-risk. This analysis uses the global model to explore a number of outstanding questions related to outbreak response after OPV cessation, including the role of key outbreak response choices (i.e., timeliness of detection and response, quality, scope, and number of rounds, vaccine type) and stockpile vaccine needs.

## Methods

This section first briefly explains essential concepts from the global model needed to interpret the outbreak response analyses in this paper, with key numerical assumption provided in Table 1. The next subsection describes the approach for the outbreak response analyses and the remaining subsections detail each outbreak response option we considered.

**Table 1:** Overview of key numerical assumptions of the models used

Assumption	Value	Source^a^
DEB model (Values based on expert review [12, 21, 22] and model calibration [9, 10] process)		
Relative contribution to transmission compared to fully susceptible, by immunity state^b^		[9, 17]
Maternally immune	0.66;0.48	
1 successful IPV dose, recent	0.74;0.41	
1 successful IPV dose, last waning stage	0.90;0.36	
2 successful IPV doses, recent	0.42;0.06	
2 successful IPV doses, last waning stage	0.81;0.13	
≥ 3 successful IPV doses, recent	0.28;0.04	
≥ 3 successful IPV doses, last waning stage	0.72;0.06	
1 LPV infection, recent	0.07;0.05	
1 LPV infection, last waning stage	0.20;0.20	
≥ 2 LPV infections or IPV and LPV (any # or order), recent	0.01;0.01	
≥ 2 LPV infections of IPV and LPV (any # or order), lastwaning stage	0.08;0.06	
Average time for maternally immune newborns to wane to fully susceptible [months]	3	[9]
Average time for other immunity states to wane from recent to fifth and last waning stage [years]		[9]
Serotypes 1 and 2	4	
Serotype 3	3	
Paralysis-to-infection ratio for WPV^c^		[9]
Serotype 1	1/200	
Serotype 2	1/2000	
Serotype 3	1/1000	
Relative R_0_ compared to serotype 1 R_0_		[9]
Serotype 2	0.9	
Serotype 3	0.75	
Relative R_0_ for OPV compared to homotypic WPV		[9, 10]
Serotype 1	0.37	
Serotype 2	0.55	
Serotype 3	0.25	
Average time to reach last of 20 reversion stages (i.e., fully-reverted VDPV, with same properties as homotypic WPV) [years]		[9, 10]
Serotype 2	1.1	
Serotypes 1 and 3	1.7	
Transmission threshold, i.e., minimal prevalence (weighed by contribution to transmission) for non-zero force-of-infection [effective infectious proportion]	5 per million	[9]
Global model [2]		
Timing of major events		
bOPV introduction for some SIAs	2010	
IPV introduction (in populations using OPV-only in 2013)	2015	
tOPV intensification (until OPV2 cessation)	2015	
OPV2 cessation (in April)	2016	
OPV13 cessation (in April)	2019	
Last year when all populations use IPV	2024	
Last full year of analytical time horizon (T_end_)	2052	
Average per-dose take rate for OPV^d^ [%]		[2, 24]
tOPV, serotype 1	35–65	
tOPV, serotype 2	60–75	
tOPV, serotype 3	27–55	
mOPV, serotype 1	45–90	
mOPV, serotype 2	60–95	
mOPV, serotype 3	45–85	
bOPV, serotype 1	42–80	
bOPV, serotype 3	42–80	
Average per-dose take rates for IPV (any serotype)^e^ [%]		[2]
Low- and lower-middle income populations	63	
Upper middle-income populations	70	
High-income populations	75	
Number of subpopulations with given R_0_ for WPV1^f^ (*N* = 710)		[2, 7]
4	20	
5	77	
6	43	
7	250	
8	90	
9	30	
10	30	
11	120	
12	20	
13	30	
Number of subpopulations with given proportion of transmissions via oropharyngeal route^g^ (*N* = 710)		[2]
0.3	290	
0.5	40	
0.6	233	
0.8	107	
0.9	40	
RI coverage and schedules	Varies^h^	[2]
Preventive SIA impact and schedules	Varies^i^	[2, 5]
Cumulative effective infections needed to trigger a potential exportation from a subpopulation (exportation threshold)	200,000	[2]
iVDPV prevalence	Varies^j^	[7]
Average time between contacts of long-term iVDPV excretors with the general population [days]	150–600	[2]
Global rate of WPV and Sabin seed strain releases from randomly determined IPV production sites [per year]	1/5	[2]
Other poliovirus releases (i.e., inadvertent OPV use, unintentional release from laboratory, intentional release)	Varies^k^	[2]

*Abbreviations*: *bOPV* bivalent oral poliovirus vaccine of serotypes 1 and 3, *DEB model* differential equation-based poliovirus transmission and OPV evolution model, *IPV* inactivated poliovirus vaccine, *iVDPV* immunodeficiency-associated vaccine-derived poliovirus, *LPV* live poliovirus, *mOPV* monovalent OPV, *OPV* oral poliovirus vaccine, *OPV## cessation* globally-coordinated cessation of OPV containing the serotype(s) indicated by ##, *PID* primary immunodeficiency disease, *RI* routine immunization, *R*_*0*_ basic reproduction number, *SIA* supplemental immunization activity, *T*_*end*_ end of the analytical time horizon (i.e., December 31, 2052), *tOPV* trivalent OPV, *WPV(1,2,3)* wild poliovirus (serotype 1, 2, or 3, respectively)

^a^Publications that list the numerical assumption and/or provide methodological details

^b^Numbers separate by semi-colons indicate contribution to a fecal-oral and oropharyngeal transmission, respectively

^c^Model assumes half of these ratios for maternally immune individuals and full and permanent protection from paralysis in all other immunity states

^d^Values vary by population and correlate with higher R_0_ values

^e^Includes priming response without seroconversion for first IPV dose

^f^R_0_ values for OPV and VDPV/WPV of each serotype follow from relative R_0_ values in top section of table

^g^Lower values correlate with higher R_0_ values

^h^See source for values by subpopulation; technical details about characterization of RI provided in [9, 25]

^i^See sources for values by subpopulation; technical details about characterization of SIAs provided in [10]

^j^Generated by discrete-event simulation model of all global PID patients [7]

^k^Depends on nature of release, income level, and time

### Global model concepts

The global model [2] integrates a characterization of the variability in conditions and mixing between populations with a differential equation-based model (i.e., the DEB model) of poliovirus transmission and OPV evolution for each population, and stochastic models of poliovirus reintroductions after OPV cessation. The global model divides the world into 710 subpopulations of approximately 10 million people, grouped into epidemiological blocks consisting of 10 subpopulations each that mix preferentially with each other. We characterize each population using pre-defined assumptions about poliovirus transmissibility (i.e., basic reproduction numbers (R_0_ values) for each virus strain, which directly relate to the assumed R_0_ for WPV1 in the population using relative R_0_ values), the relative importance of fecal-oral and oropharyngeal transmission (which affects the impact of IPV use on transmission), OPV and IPV take rates, immunization history and intensity going forward (e.g., RI coverage, SIA frequency and quality), surveillance quality (i.e., number of cumulative paralytic cases to trigger detection of an outbreak), and age-heterogeneity in mixing.

All global model runs assume the same run-up until 2013, after which the results depend on long-term poliovirus risk management policies and random events. Long-term poliovirus risk management policies run through 2052 and include OPV cessation with different durations of IPV use. The main policy option remains consistent with the Global Polio Eradication Initiative Strategic Plan 2013–2018 [1] and assumes globally-coordinated cessation of serotype 2-containing OPV (OPV2 cessation) in 2016, globally-coordinated cessation of serotype 1- and  3-containing OPV (OPV13 cessation) in 2019, and 5 subsequent years in which all populations use at least one IPV RI dose (i.e., IPV5).

cVDPV outbreaks occur deterministically in the model depending on population immunity at and beyond OPV cessation, but all other potential poliovirus reintroductions (from iVDPVs, IPV productions sites, inadvertent OPV use, or other unintentional or intentional releases) occur randomly. A discrete-event simulation model estimates the prevalence of primary immunodeficiency disease patients with long-term iVDPV infections, including any patients infected with OPV used for outbreak response after OPV cessation [7]. Potential poliovirus exportations occur whenever a threshold number of infections accumulate in a subpopulation as a result of exposure to the outbreak virus or OPV used to respond to it, with the destination populations determined randomly.

Transmission of a poliovirus in the DEB model only occurs when the effective prevalence of that poliovirus resides above the transmission threshold. Thus, an outbreak may die out within a subpopulation if natural immunity derived by the outbreak virus and/or immunity derived by the outbreak response drives the prevalence below the transmission threshold. Similarly, depending on population immunity to poliovirus transmission in a polio-free subpopulation, potential poliovirus reintroductions or importations may or may not generate enough initial transmissions to exceed the transmission threshold and start to circulate at the subpopulation level. We model this by specifying a function for the probability that an introduction becomes “effective”. Specifically, the probability increases with the net reproduction number (R_n_), defined as the average number of secondary infections generated by each new infection, taking into account both the R_0_ of the virus and the population immunity level and mixing [8]. If R_n_ > 1, then the poliovirus can circulate in a population, and the growth rate of the outbreak increases as R_n_ increases. To calculate R_n_, we account for the relative R_0_ for the WPV serotype, the serotype-specific relative R_0_ for OPV compared to homotypic fully-reverted VDPV (i.e., assuming the same R_0_ as homotypic WPV, which implies relative values of 1:0.9:0.8 for VDPV and WPV serotypes 1, 2, and 3, respectively), and the seasonal variation in poliovirus transmissibility [2, 9, 10].

Due to random events, each stochastic iteration of the global model produces a different possible future, and therefore we based economic estimates of long-term poliovirus risk management policies on the average of a set of 100 stochastic iterations [2]. In the event of uncontrolled outbreaks, paralytic polio cases will continue to accumulate after OPV cessation. If this leads to at least 50,000 paralytic polio cases, then the global model assumes that all populations that used OPV as of 2013 would resume OPV use, which we refer to as OPV restarts. In this study, we assume that all OPV restarts resume OPV in RI but not in SIAs.

### Analytical approach

Table 2 lists the outbreak response assumptions for all policies analyzed in the global model base case [2] and alternative choices explored in this study. All analyses assume the same main long term global poliovirus risk management policy of at least 5 years of IPV use after OPV13 cessation in all populations, but they vary the outbreak response assumptions. The analyses repeat one or more of the 100 global model iterations for each outbreak response choice. Table 2 does not include choices about the duration of each outbreak response SIA (oSIA), which we fixed at 5 days, or the target age groups (i.e., 0–4 years between 0 and 4 years after homotypic OPV cessation, 0–9 years between 5 and 9 years after homotypic OPV cessation, 0–14 years between 10 and 14 years after homotypic OPV cessation, etc.), which we did not vary in this analysis. As shown in the last two columns of Table 2, we examined the impact of each outbreak response choice by looking at the behavior for specific examples, or by examining global model outputs for the full set of 100 iterations, or both, depending on the outcomes of interest and computational resource requirements. When examining outputs for the full set of iterations, we consider all iterations affected by each change from the set of 100 iterations for the IPV5 policy (Table 2). For example, reducing the duration of mOPV use for oSIAs after homotypic OPV cessation from 5 to 3 years only affects 77 iterations in which at least one outbreak response occurs from 3 to 5 years after homotypic OPV cessation.

**Table 2:** Outbreak response choices considered

Outbreak response choice	Base case	Alternative(s)	Iterations considered	Outcomes of interest
Initial number of oSIAs^a^	4 (R_0_ < 12)	3, 4, 5, 6, or 7	1 selected iteration	Behavior
	6 (R_0_ ≥ 12)			
oSIA impact level^b,c^	B	A, B, or C	1 selected iteration	Behavior
Interval between oSIAs (days)^c^	30	15	1 selected iteration	Behavior
Mixed IPV/OPV use (ring)	None	mOPV (outbreak subpopulation); IPV (other subpopulations in block)	Selected iterations	Behavior, mOPV exportations, new iVDPV excretors, OPV restarts
Duration of mOPV use after homotypic OPV cessation (years)	5	3, 10, 20, or through T_end_	All affected	Effective mOPV exportations; new iVDPV excretors; OPV restarts
Geographical scope (minimum R_0_ to trigger block-wide response)	10	8 or 13	All affected	Effective mOPV exportations; new iVDPV excretors; OPV restarts
Response delay (days)^d^ and detection threshold^e^	Delay 45 and threshold variable (initial detection) or delay 30 and threshold 1 (oSIAs ongoing in block)	Delay always 30, 45, or 50 and threshold always variable	All affected	Behavior; OPV restarts
Serotype 2 vaccine between OPV2 and OPV13 cessation	Serotype 2 mOPV	tOPV, IPV	All affected +1 iteration without tOPV intensification	Population immunity; number and size of outbreak; OPV restarts
Finite mOPV stockpile	Unlimited stockpiles	100 million filled and 400 million bulk doses of each mOPV serotype	All affected	Behavior; OPV restarts

*Abbreviations*: *IPV* inactivated poliovirus vaccine, *iVDPV* immunodeficiency-associated vaccine-derived poliovirus; mOPV, monovalent OPV, *OPV##* cessation, globally-coordinated cessation of OPV containing the serotype(s) indicated by ##, *OPV* oral poliovirus vaccine, *oSIA* outbreak response supplemental immunization activity, *R*_*0*_ basic reproduction number, *T*_*end*_ end of the analytical time horizon (i.e., December 31, 2052), *tOPV* trivalent OPV

^a^Considered jointly with oSIA impact level and interval between oSIAs

^b^Key for SIA impact levels: A = true coverage of 0.5 and repeated missed probability if 0.8; B = true coverage of 0.8 and repeated missed probability if 0.7; C = true coverage of 0.95 and repeated missed probability if 0.5

^c^Considered jointly with initial number of oSIAs

^d^Time between detection and first day of first oSIA

^e^Cumulative number of paralytic cases per 10 million people to trigger a detection

### Initial number of oSIAs. oSIA impact level, and interval between oSIAs

Upon outbreak detection in a subpopulation, the base case outbreak response strategy conducts between 4 and 6 high-quality oSIAs, depending on the subpopulation R_0_, with a block-wide response if the detection occurs in a subpopulation with an R_0_ of 10 or more (Table 2). An additional series of 4–6 oSIAs may occur in the event of a detection of a case after the last oSIA in a series. We characterize oSIA quality using three oSIA impact levels that represent different combinations of true coverage (i.e., the overall proportion of targeted individuals receiving a dose), and repeated missed probability (i.e., the proportion of individuals targeted but missed by the previous SIA who again do not receive a dose). To explore how the choice of the initial number of oSIAs interacts with the quality of the oSIAs, we simultaneously varied both parameters and focused on their impact on the behavior of an outbreak that occurs in a block with a very high R_0_ of 13 following a serotype 1 iVDPV (iVDPV1) introduction. For the same outbreak, we also considered the interaction between the initial number of oSIAs and the interval between each oSIA in a series, which equals 30 days for the base case.

### Mixed IPV/OPV use (ring)

We selected two stochastic iterations to explore the potential strategy of conducting outbreak response with mOPV in the outbreak population, and a ring of IPV in surrounding populations, which we operationalized in the model as mOPV use in any subpopulation with a detected case and IPV use in all other subpopulations of the same block.

### Duration of mOPV use after homotypic OPV cessation

The base case outbreak response strategy assumes mOPV use only for 5 years after global homotypic OPV cessation to avoid reintroducing large amounts of LPV at a time of globally much-decreased population immunity, with IPV use for any subsequent oSIAs. We explored the impact of different durations of mOPV use on effective mOPV exportations, new iVDPV infections, and OPV restarts (Table 2). For further context on the time interval during which mOPV can be safely used for oSIAs, we report for the base case outbreak response strategy the fraction of subpopulations for which the R_n_ of OPV of each serotype exceeds 1 as a function of time. As R_n_ increases, both the probability of an effective mOPV introduction as a result of an mOPV exportation and the consequences of an effective mOPV introduction increase.

### Geographical scope

We consider the impact of the minimum R_0_ that triggers a block-wide response on effective mOPV exportations, new iVDPV infections, and OPV restarts (Table 2).

### Response delay and detection threshold

In the event of a subpopulation-specific outbreak response, the base case strategy assumes that all other subpopulations in the same block remain on “high alert” for surveillance and response until the last oSIA in the block, which leads to a low detection threshold of one paralytic case and a shorter response delay of 30 instead of 45 days (Table 2). We considered the impact of not moving the neighboring subpopulations to “high alert” and also varied the response delay between 30 and 60 days while keeping the detection threshold unaffected by the ongoing oSIAs.

### Serotype 2 vaccine

In the event of serotype 2 outbreaks between OPV2 cessation and OPV13 cessation, using trivalent OPV (tOPV) instead of mOPV2 could raise population immunity to transmission for serotypes 1 and 3 before OPV13 cessation. Although logistical and regulatory constraints may complicate tOPV use after OPV2 cessation, we explored the use of tOPV for outbreak response instead of mOPV2 by characterizing the changes in population immunity to transmission for all 3 serotypes, and the number of OPV restarts. Given current discussions to use IPV in response to cVDPV2 outbreaks, we further considered the option of using IPV instead of mOPV2 or tOPV for outbreaks that occur between OPV2 and OPV13 cessation. To explore cVDPV2 outbreak and population immunity behavior for these vaccine choices, we ran the model without tOPV intensification prior to OPV2 cessation and considered the resulting outbreak.

### Stockpile choices

The base case response assumes no constraints on the amount of vaccine available for oSIAs from a stockpile. In reality, the GPEI plans to hold approximately 100 million filled and 400 million bulk mOPV doses of each serotype. To further stockpile planning, we report the expected mOPV and IPV stockpile needs for each serotype as a function of time after homotypic OPV cessation for the base case outbreak response strategy based on all 100 iterations. To estimate stockpile vaccine needs, we multiply the targeted population of each oSIA by an effective wastage factor that conservatively accounts for demographic uncertainty [2, 5]. We further explore the potential consequences of the currently planned mOPV stockpile for all iterations in which a stock-out would occur given the currently planned filled and bulk mOPV stocks, with all other assumptions as in the base case response strategy. To determine when a stock-out of readily available filled mOPV would occur, we assume that new orders to convert bulk to filled vaccine occur on the first day of each oSIA, leading to a one year filling delay with a “pipeline delay”, so that all newly-ordered filled vaccine arrives exactly one year after placing the order [11]. For simplicity, these analyses conservatively assume no prioritization of the mOPV in case of a shortage and assume that any IPV oSIAs will not occur until after closure of the 5-year mOPV use window. For all of the analyses in Table 2, we consider how the stockpile needs change in addition to the other outcomes of interest listed in Table 2.

## Results

This section presents the results related to each outbreak response choice in separate subsections.

### Initial number of oSIAs. oSIA impact level, and interval between oSIAs

**Fig. 1:**
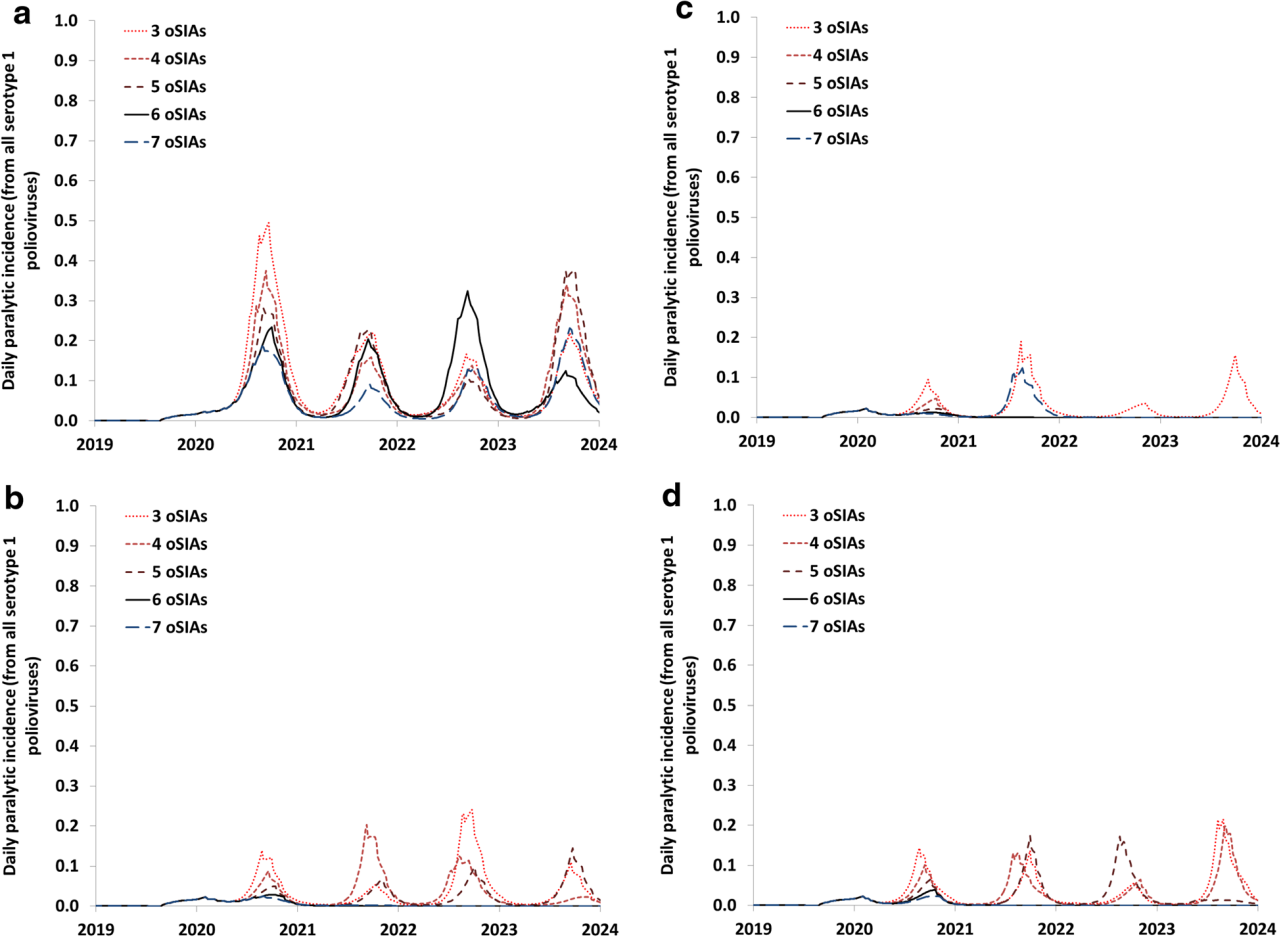
Outbreak and response behavior for different initial number of outbreak response supplemental immunization activities (oSIAs), oSIA quality, and oSIA interval assumptions for an outbreak in a high basic reproduction number population, showing the incidence in the block of the initial outbreak. **a** oSIA impact level A (i.e., true coverage of 0.5 and repeated missed probability^*^ of 0.8). **b** oSIA impact level B (i.e., true coverage of 0.8 and repeated missed probability of 0.7). **c** oSIA impact level C (i.e., true coverage of 0.95 and repeated missed probability of 0.5). **d** oSIA impact level B (i.e., true coverage of 0.8 and repeated missed probability of 0.7), but with 15 instead of 30 days between oSIAs. ^*^ The repeated missed probability represents the proportion of targeted individuals missed by an SIA who were targeted and missed by the previous SIA [10]

Figure 1 shows the outbreak behavior for different combinations of quality and initial quantity of oSIAs and for different intervals between oSIAs. The outbreak in this selected iteration originates from an iVDPV1 introduction soon after OPV13 cessation in a block with the highest R_0_ in the global model (i.e., R_0_ = 13 for WPV1). Due to the high R_0_, the model assumes a block-wide outbreak response. Figure 1a shows that in this population, low quality oSIAs (i.e., true coverage of 50 % per round and repeated missed probability of 80 %) do not control the outbreak, even if the response involves 7 oSIAs at 30-day intervals in each series. Failure to contain the outbreak eventually results in exportations to other blocks with low population immunity to transmission and a need to restart OPV use after more than 50,000 paralytic cases accumulated since 2016. For the base case response strategy quality level (i.e., true coverage of 80 % per round and repeated missed probability of 70 %), control of the outbreak becomes possible with 6 or more oSIAs per series but not with 5 or fewer oSIAs per series (Fig. 1b). However, the initial series does not prevent a second peak later in 2020, and thus die-out occurs only after two series of 6 or 7 oSIAs. Further increasing the quality (i.e., true coverage of 95 % per round and repeated missed probability of 50 %) significantly improves performance, with two series of 4 or more oSIAs successfully containing the outbreak. Figure 1d shows that shortening the interval between oSIAs in a series from 30 to 15 days somewhat affects the kinetics of the outbreak compared to Fig. 1b, but does not change the minimum of 6 oSIAs needed per series to control the outbreak.

### Mixed IPV/OPV use (ring)

**Fig. 2:**
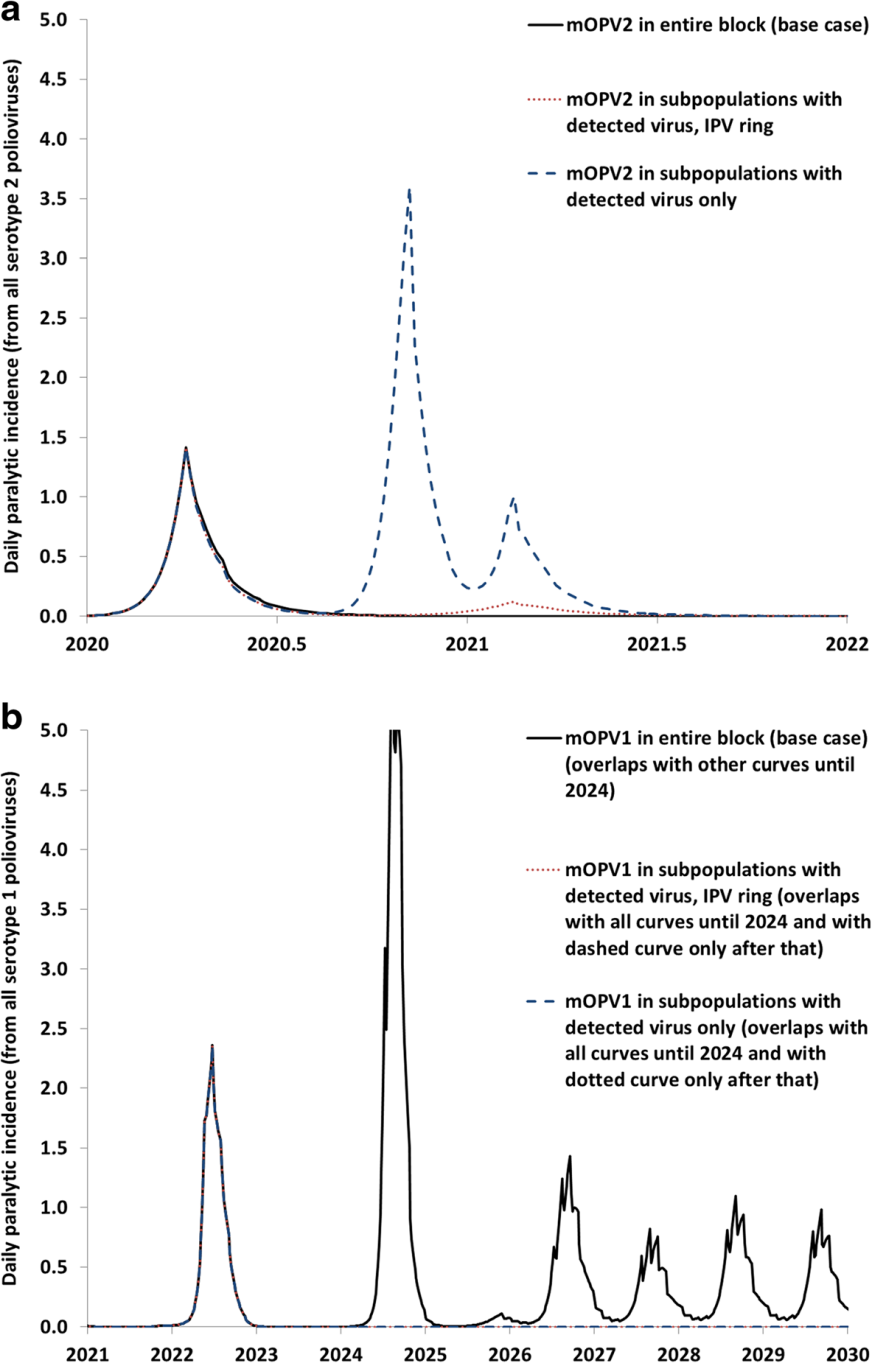
Two contrasting examples of serotype 1 and 2 immunodeficiency-associated vaccine-derived poliovirus (iVDPV1 and iVDPV2, respectively) outbreaks with various outbreak response vaccine choices for subpopulations that share a block with subpopulations that detected a case , including a ring with inactivated poliovirus vaccine (IPV), showing the incidence in the block of the initial outbreak. **a** Outbreak following an iVDPV2 introduction in a block with a basic reproduction number (R_0_) of 10, resulting in a serotype 2 monovalent oral poliovirus vaccine (mOPV2) exportation outbreak in another block for the strategy that responds only in subpopulations with detected virus using mOPV2. **b** Outbreak following an iVDPV1 introduction in a block with an R_0_ of 11, resulting in a new iVDPV1 excretor and virus reintroduced for the strategy of serotype 1 monovalent oral poliovirus vaccine (mOPV1) in the entire block

Figure 2 shows two contrasting examples of outbreaks involving different ring strategies. The base case response strategy assumes block-wide response with mOPV following detection of the outbreak virus in any of its subpopulations. The IPV ring strategy assumes mOPV use in the subpopulation that detected the outbreak and IPV use in the other 9 subpopulations of its block, while the third strategy assumes no oSIAs in other subpopulations until they detect virus. In the first example, the base case, block-wide mOPV response strategy contains the outbreak in the subpopulation that experiences the introduction (Fig. 2a, solid curve). In contrast, with a subpopulation-specific response, exportations of the outbreak virus to other subpopulations in the same block can take off, leading to new outbreaks that trigger further subpopulation-specific oSIAs (Fig. 2a, dashed curve). In this stochastic realization of exportation events, a serotype 2 mOPV (mOPV2) virus used in one of the secondarily affected subpopulations establishes transmission in a block with a WPV1 R_0_ of 13 in which the population immunity to transmission decreased so much after OPV2 cessation that it can support transmission of even the attenuated mOPV2 virus. With successive chains of transmission, this virus eventually evolves to a cVDPV2 and triggers an OPV restart. Using mixed mOPV2 in the outbreak subpopulation and IPV in the other 9 subpopulations prevents this from occurring (Fig. 2a, dotted curve). Although the IPV oSIAs do not completely prevent the secondary outbreaks they keep the outbreaks smaller and fewer in number. Thus, in this example, the IPV ring works not because it contains the mOPV2 used in the initial response, but because it prevents the need for some secondary subpopulation-specific oSIAs that otherwise become problematic.

In the second example, a different problem occurs as a result of mOPV use during oSIAs. The base case, block-wide mOPV1 response strategy to an iVDPV1 outbreak successfully controls the outbreak (Fig. 2b, solid curve). However, in the realization of the risks, a patient with PID and prone to long-term poliovirus infection acquires an mOPV1 infection in one of the subpopulations not directly affected by the outbreak that conducts a pre-emptive mOPV1 oSIA as part of the block-wide response strategy. This new iVDPV1 excretor re-introduces the virus years later, at which point the model no longer assumes mOPV1 availability for outbreak response. The subsequent IPV oSIAs fail to control the outbreak, and thus this iteration ultimately leads to one of the two OPV restarts that we observed for the base case. In this example, the subpopulation-specific outbreak response (with or without the IPV ring) suffices to control the outbreak and avoids the mOPV1 infection that generates the new iVDPV1 excretor. We emphasize that both examples in Fig. 2 depend on rare stochastic events (i.e., initial iVDPV introductions, effective OPV exportations, and new iVDPV infections and introductions) that we did not observe in other iterations with the base case. However, the possible behavior in Fig. 2 illustrates the dilemma associated with vaccine type and oSIA scope decisions, with mOPV carrying some risk of new events and IPV oSIAs carrying a substantial risk of failing to control outbreaks.

### Duration of mOPV use after homotypic OPV cessation

**Fig. 3:**
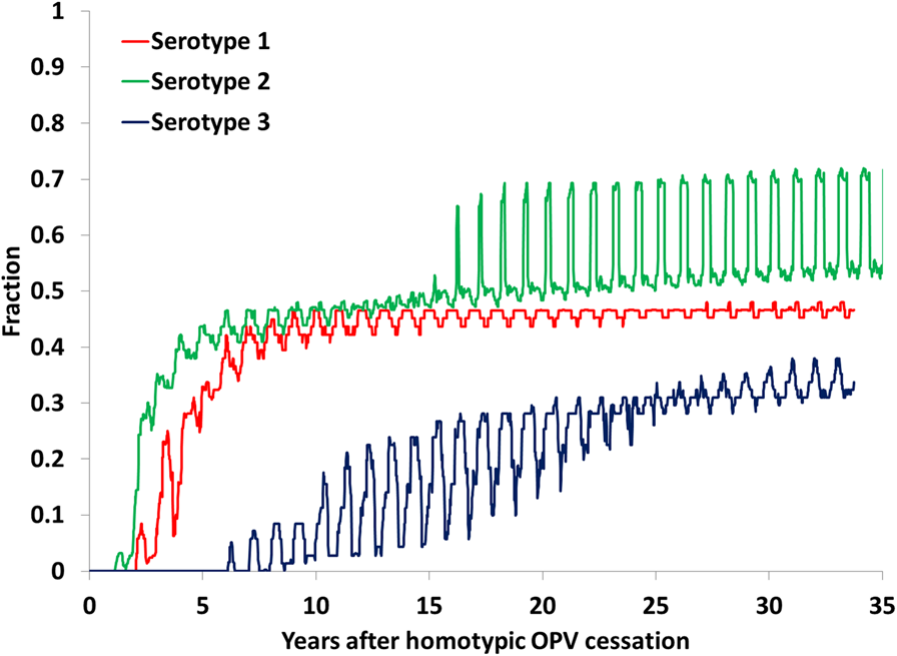
Proportion of subpopulations (*n* = 710) with a net reproduction number (R_n_) of oral poliovirus vaccine (OPV) of more than 1 for a global model iteration with no outbreaks with the global policy of at least 5 years of inactivated poliovirus vaccine in all populations for 5 years after OPV cessation of the last serotype

In the context of assessing the risk of exportation of mOPV used during oSIAs, Fig. 3 shows the proportion of all subpopulations in the model with low enough population immunity to transmission to support transmission of mOPV of each serotype (i.e., R_n_ > 1 for OPV) as a function of time since homotypic OPV cessation. The results differ by type primarily because of the difference in assumed relative R_0_ values of OPV compared to homotypic WPV or VDPV (i.e., OPV2 > OPV1 > OPV3) [9, 10, 12]. The results oscillate because of seasonal changes in R_0_ and therefore in R_n_ [2, 13, 14]. Figure 3 suggests that within 2–3 years of OPV cessation, some transmission may occur in the event of imported mOPV2 and mOPV1 in some subpopulations, with little risk of mOPV3 establishing transmission for at least 5 years after OPV cessation. However, with R_n_ only slightly above 1 for some part of the year, such transmission may not result in a VDPV outbreak. Moreover, potential mOPV exportations to other blocks occur relatively rarely, and we assume that chance determines whether potential exportations effectively establish transmission beyond the initial contacts [2].

Table 3 includes results related to mOPV exportations for the various outbreak response scenarios, summed over all 100 iterations. For the base case, we find that potential mOPV-related exportations occur regularly (i.e., approximately 36 per stochastic iteration on average). Given that most of these occur during the first few years after homotypic OPV cessation when population immunity remains high in most subpopulations (Fig. 3), only approximately 10 % of them result in effective mOPV-related virus reintroduction. Due to the assumed highly preferential mixing within blocks [2], over 95 % of the effective exportations remain in the same block. The block-wide response strategy in blocks with an R_0_ of 10 or more in the base case helps prevent most of these exportations from evolving to VDPVs, while in lower-R_0_ blocks the R_n_ values for OPV-related viruses typically remain low enough to prevent transmission. For the 14 effective exportations to other blocks that occurred in the 100 iterations with the base case response strategy (Table 3), none led to any outbreaks because the R_n_ values all remained close to 1. The use of mOPV for oSIAs also creates a risk of newly infecting PID patients with potential long-term excretion, which occurred in the simulations approximately once per iteration on average. However, the majority of these occur in populations with higher R_0_ values, which account for most mOPV oSIA doses and in which survival of PID patients remains shortest [7]. Therefore, newly infected PID patients only generate few potential iVDPV introductions (i.e., on average approximately one per excretor), and most occur soon after the outbreak response when population immunity to transmission remains high, which prevents the potential iVDPV introductions from becoming effective introductions or outbreaks. However, 10 of the 22 effective iVDPV introductions in the base case resulted in a new outbreak of cases that required a response. In one iteration, an outbreak due to a new iVDPV excretor beyond the 5-year window of mOPV use for outbreak response results in uncontrolled outbreaks and an eventual OPV restart (see Fig. 2b). The two iterations with an OPV restart by definition result in very large numbers of expected cases, and therefore drive the average expected number of cases based on all 100 iterations, which equals approximately 15,000.

**Table 3:** Impact of outbreak response choices on effective mOPV exportations, new iVDPV excretors, and OPV restarts

Outbreak response choice or assumption	Number of affected iterations	mOPV exportations (totals from 100 iterations)			PID patients infected with OPV used during oSIAs (totals from 100 iterations)				OPV restarts	Expected paralytic cases 2013–2052 (after OPV cessation)^a^		
		Potential expor-tations	Effective reintro-ductions (to other blocks)	Out-breaks	Newly infected long-term excretors	Potential iVDPV reintro-ductions	Effective iVDPV reintro-ductions	Out-breaks		No OPV restart	OPV restart	All
Base case	N/A	3,618	312 (14)	0	117	96	22	10	2	340	720,000	15,000
Duration of mOPV use after homotypic OPV cessation (years)												
- 3	77	3,141	255 (11)	0	84	57	15	7	10^b^	240	850,000	85,000
- through T_end_	30	4,153	364 (18)	0	136	119	23	19	0	370	-	370
Minimum R_0_ to trigger block-wide response												
- 8	45	5,709	479 (23)	0	173	151	34	16	2^b^	370	610,000	13,000
- 13	43	1,323	155 (12)	1	64	66	15	7	4	370	1,000,000	40,000
Response delay (days)^c^												
- Always 30	96	3,521	300 (15)	0	106	99	23	10	2	240	530,000	11,000
- Always 45	92	3,620	311 (14)	0	117	96	22	10	3	620	1,000,000	29,000
- Always 60	96	3,936	335 (18)	0	133	106	23	10	6	640	1,100,000	64,000
oSIA vaccine between OPV2 and OPV13 cessation for serotype 2 outbreaks												
- tOPV	36	3,634	315 (5)	0	117	96	21	10	2	340	720,000	15,000
- IPV	36	3,586	317 (14)	1	121	96	22	11	3	360	740,000	22,000
Finite mOPV stockpile	27	3,962	347 (19)	0	166	102	24	10	7^b^	1,300	770,000	55,000

*Abbreviations*: *IPV* inactivated poliovirus vaccine, *iVDPV* immunodeficiency-associated vaccine-derived poliovirus; mOPV, monovalent OPV, *OPV* oral poliovirus vaccine, *OPV## cessation* globally-coordinated cessation of OPV containing the serotype(s) indicated by ##, *oSIA* outbreak response supplemental immunization activity, *PID* primary immunodeficiency disease, *R*_*0*_ basic reproduction number, *T*_*end*_ end of the analytical time horizon (i.e., December 31, 2052), *tOPV* trivalent OPV

^a^Does not include a total of approximately 1,000 expected WPV, VAPP, and cVDPV cases that occur before OPV cessation of each serotype [2]

^b^One additional iteration had ongoing LPV transmission at T_end_ without having accumulated 50,000 cases since 2016

^c^All alternative choices assume non-adaptive surveillance quality (detection threshold) and response delay in the event of a subpopulation-specific response

Reducing the window of mOPV use from 5 to 3 years after homotypic OPV cessation led to a clear reduction in the number of mOPV exportations and newly infected iVDPV excretors and associated events. However, with no mOPV use allowed to respond to new outbreaks between 3 and 5 years after homotypic OPV cessation or outbreaks not controlled within the first 3 years, control of these outbreaks (i.e., with IPV oSIAs) becomes much more difficult. In this case, 8 additional iterations resulted in an OPV restart for the 3-year mOPV use window, which increased the average expected number of cases from 15,000 to 85,000. Conversely, allowing mOPV use for oSIAs through the end of the model time horizon (i.e., T_end_) substantially increased the number of mOPV exportations and new iVDPVs. Although the population immunity levels over 5 years after OPV cessation of each serotype support mOPV transmission in a large fraction of all subpopulations in the model (Fig. 3), remarkably none of the approximately 360 effective mOPV exportations or 23 effective iVDPV introductions resulted in uncontrolled outbreaks. This reflects the location of the majority of these effective exportations and introductions occurring in subpopulations that benefit from higher population immunity due to recent mOPV oSIAs. In the event of outbreaks not controlled in the first subpopulation, mOPV exportations sometimes occurred but resulted in no independent outbreak events because the outbreak virus got exported before or soon after the OPV virus and caused cases before OPV evolved to a cVDPV. Moreover, for any effective introductions that do lead to outbreaks, including for the two iterations with an OPV restart for the base case, the use of mOPV in response to late outbreaks facilitates rapid control and containment of these outbreaks. Intuitively, an aggressive outbreak response strategy that limits propagation of a given outbreak to one new block or fewer on average leads to the eventual full control of the outbreak. For mOPV use in oSIAs through T_end_, this occurs in the global model despite the risk of mOPV exportations and new iVDPV excretors. Consequently, we observed no OPV restarts and the lowest average expected number of cases for this outbreak response strategy among all considered options. However, we caution that the global model does not characterize the mixing patterns at the border of the mOPV oSIA target population where exportations to other areas may occur much more frequently than the average frequency of exportations between populations.

### Geographical scope

Increasing the geographical scope of the outbreak response to include a block-wide response for all outbreaks in populations with an R_0_ of 8 or more (instead of 10 or more) led to more mOPV exportations and new iVDPV introductions compared to the base case. However, these did not result in uncontrolled outbreaks or new OPV restarts. To the contrary, the expanded scope reduces the number of cases for the two iterations with an OPV restart for the base case, which resulted in a relatively small but notable reduction in the average expected number of cases. Reducing the geographical scope of the outbreak response reduced the risk of any outbreaks due to mOPV exportations or new iVDPV excretors, but led to a failure to control outbreaks in 2 additional iterations and thus a higher average expected number of cases. One of these OPV restarts related to a mOPV exportation that eventually caused new cVDPV outbreaks after the 5-year period of mOPV use, as discussed in the context of Fig. 2b.

### Response delay and detection threshold

The response delay affects the ability to contain outbreaks before further spread. Therefore, we observed an increase in the number of OPV restarts and average expected number of cases as we increased the response delay. The effect on mOPV exportations and new iVDPV excretors remains moderate, with a slight increase in these events for a slower response because the slower response requires more oSIAs due to failures to prevent outbreak virus exportations or to control the outbreak after the initial oSIA series. Reducing the response delay from 45 to 30 days significantly decreased the average expected cases for both iterations with and without an OPV restart. The choice of oSIA vaccine for serotype 2 outbreaks affects population immunity to transmission for the other two serotypes, but did not much affect the overall results based on 36 affected iterations in Table 3. For the IPV option, this relates to the assumption that the oSIA strategy reverts to mOPV2 after OPV13 cessation, which allows control of most uncontrolled serotype 2 outbreaks. Nevertheless, in one iteration, the use of IPV for oSIAs before OPV13 cessation resulted in uncontrolled outbreaks and an OPV restart, which increased the average expected number of cases for this strategy. In another iteration, IPV oSIAs failed to control a serotype 2 outbreak before switching to mOPV2 oSIAs after OPV13 cessation, which resulted in an mOPV2 exportation that required an outbreak response but did not lead to an eventual OPV restart.

### Serotype 2 vaccine

**Fig. 4:**
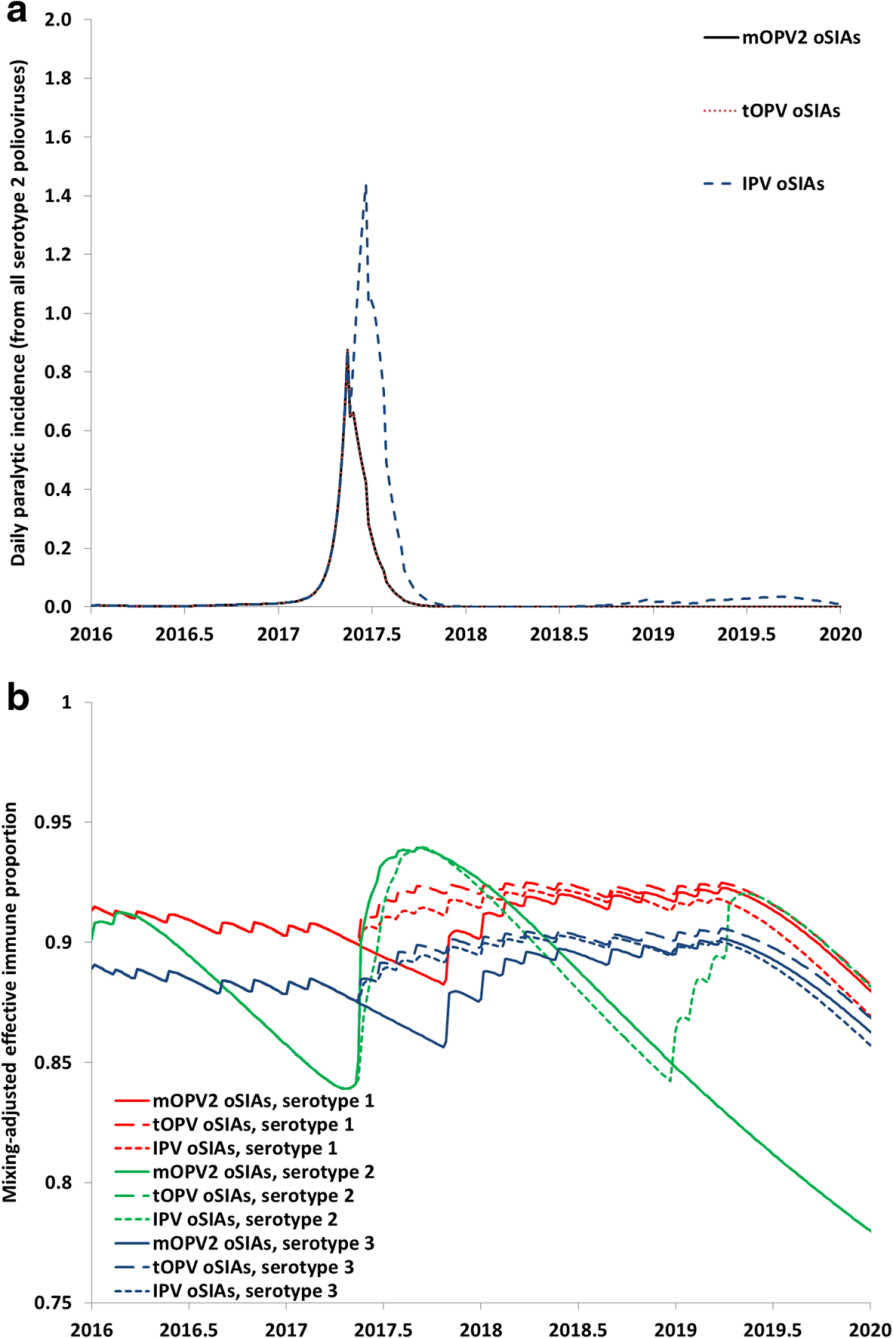
Example of outbreak response supplemental immunization activity (oSIA) choices to a serotype 2 circulating vaccine-derived poliovirus (cVDPV2) outbreak that occurs after serotype 2 oral poliovirus vaccine (OPV) cessation without prior triavelent OPV (tOPV) intensification, using serotype 2 monovalent OPV (mOPV2), tOPV, or inactivated poliovirus vaccine (IPV). **a** Incidence of paralytic poliomyelitis cases (in the block of the cVDPV2 outbreak). **b** Population immunity to transmission for all 3 serotypes, expressed as the mixing-adjusted immune proportion (EIPM) in the subpopulation of the cVDPV2 outbreak

Figure 4 illustrates the behavior for the different serotype 2 oSIA vaccine choices, based on a run with insufficient tOPV intensification that results in a cVDPV2 outbreak within a year after OPV2 cessation (and no other risks included in Fig. 4) [2, 5]. Outbreak responses with tOPV or mOPV2 remain equivalent in terms of the ability to rapidly control the outbreak due to assumed equal average per-dose take rates for serotype 2 in the affected population (Fig. 4a) [2]. In contrast, the 4 IPV oSIAs in the outbreak block fail to fully control the outbreak, which leads to a second outbreak wave in 2019 that eventually gets controlled by assumed mOPV2 use for oSIAs following OPV13 cessation in 2019. Figure 4b shows the clear benefit of tOPV over mOPV2 with respect to sustaining high population immunity to serotype 1 and 3 transmission for the duration of the outbreak response, which the model assumes suspends any planned preventive SIAs with serotype 1 and 3 bivalent OPV. IPV oSIA also sustain some population immunity to transmission, but not as much as tOPV, and this benefit trades off poorly against the inability of IPV to control the cVDPV2 outbreak that triggered its use. The increase in population immunity to serotype 2 transmission for the IPV oSIAs curve during 2019 reflects the second outbreak wave that occurs with that outbreak response strategy.

### Stockpile choices

**Fig. 5:**
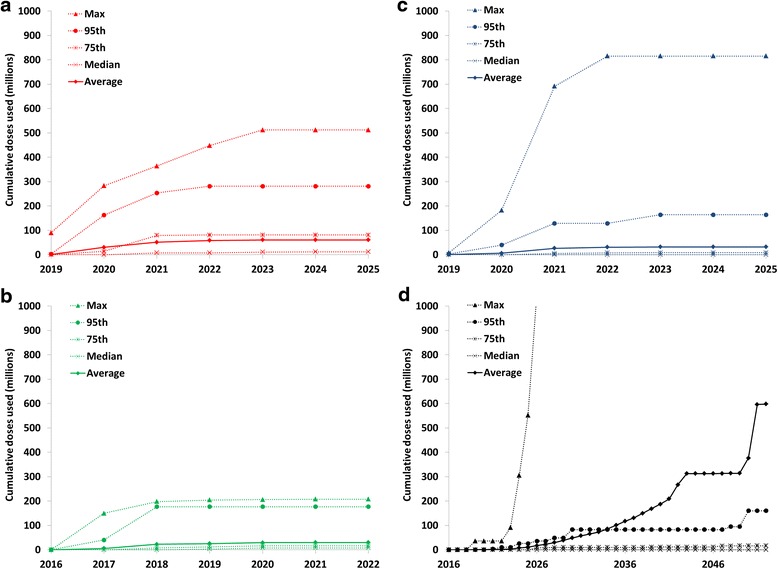
Outbreak response supplemental immunization activity (oSIA) vaccine usage for the base case outbreak response strategy, based on 100 iterations with the global policy of at least 5 years of inactivated poliovirus vaccine (IPV) in all populations for 5 years after oral poliovirus vaccine (OPV) cessation of the last serotype (note change in x-axis scales). **a** Summary statistics of serotype 1 monovalent OPV oSIA needs over time. **b** Summary statistics of serotype 2 monovalent OPV oSIA needs over time. **c** Summary statistics of serotype 3 monovalent OPV oSIA needs over time. **d** Summary statistics of IPV oSIA needs over time

Figure 5 shows the cumulative oSIA vaccine needs based on all 100 iterations of the global model with IPV5 and the base case response strategy and accounting for significant assumed wastage. The averages and medians in Fig. 5 remain well below the currently planned 100 million filled doses for each mOPV serotype. However, 32 stochastic iterations require more than 100 million mOPV doses for at least one serotype (i.e., 23 for mOPV1, 12 for mOPV2, 8 for mOPV3) and the maxima for mOPV1 and mOPV3 both exceed the total planned bulk and filled stockpile of 500 million doses. While these represent outliers from the distribution, they suggest some risk of insufficient stockpiled vaccine to meet the expected demand for the base case response strategy. With respect to the IPV stockpile vaccine needs, we similarly observe one outlier that involves repeated IPV oSIAs that fail to control an outbreak but do prevent the total number of cases from reaching the 50,000 threshold that would trigger OPV restart for many years. While this outlier explains the high average IPV oSIA needs, Fig. 5d also shows non-zero expected IPV needs for oSIAs over the long-term at the 75^th^ percentile. This suggests a relatively high chance of some IPV oSIA demand and the need to build a global IPV stockpile for outbreak response, which could be operationalized as a rotating IPV stock in the long-term IPV supply chain [15], especially because mOPV represents an increasingly risky oSIA option as the time since OPV cessation increases and the mOPV stockpile will represent a finite resource [16].

In the event of cumulative vaccine needs of more than the currently planned 100 million filled mOPV doses, the occurrence of any stock-outs depends on how rapidly the mOPV demand unfolds and how long it takes to convert bulk to filled mOPV (i.e., the filling delay). Assuming a one year filling delay, we observed stock-outs of at least one mOPV serotype in 27 of 100 iterations with the base case response strategy.

**Fig. 6:**
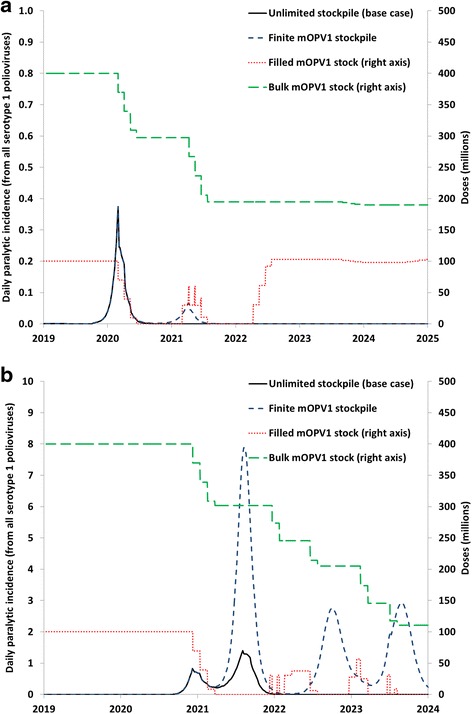
Two examples of model behavior with assumed unlimited vs. finite monovalent oral poliovirus vaccine (OPV) stockpile for the base case outbreak response strategy and the global policy of at least 5 years of inactivated poliovirus vaccine in all populations for 5 years after oral poliovirus vaccine (OPV) cessation of the last serotype. **a** Outbreak following a serotype 1 immunodeficiency-associated vaccine-derived poliovirus (iVDPV1) introduction in a block with a basic reproduction number (R_0_) of 11, with stock-out resulting in a second outbreak wave but ultimate outbreak control. **b** Outbreak following an iVDPV1 introduction in a block with an R_0_ of 12, with stock-out resulting in a failure to control the outbreak and an eventual OPV restart (note change in y-axis scale)

Figure 6 shows two examples of the potential consequences of mOPV1 stockouts, both involving block-wide oSIAs in response to iVDPV1 introductions in relatively high-R_0_ blocks. In Fig. 6a, a stock-out occurs for the last of 4 block-wide mOPV1 oSIAs because the estimated total doses within the series of oSIAs over a period of approximately 4 months exceeds 100 million. The stock-out results in reduced coverage during the last oSIA, and consequently the virus sustains a low level of transmission until it leads to another outbreak peak the following year (dashed blue curve in Fig. 6a). This second peak does not occur with an infinite stockpile (solid black curve in Fig. 6a). With higher population immunity to transmission during the second outbreak, the triggered new mOPV1 oSIA series controls the outbreak. The filled mOPV1 stock again goes to zero, but sufficient bulk mOPV1 remains to replenish the stock and no further mOPV1 oSIA demands occur before the filled stock restores to 100 million mOPV1 doses. Due to a small iVDPV1 outbreak in another subpopulation with much lower R_0_, some more mOPV1 gets used before the 5-year mOPV1-use window closes, with little impact on the filled mOPV1 stock. In contrast, in Fig. 6b, a second outbreak peak occurs much sooner than after the initial mOPV1 oSIA series failed to control the outbreak in Fig. 6a. With unlimited filled mOPV1 available, the second series of oSIAs controls the outbreak (solid black curve in Fig. 6b). However, with 100 million filled mOPV1 doses and a one year filling delay, the second oSIA series cannot take place, leading to a very large outbreak in the second year (dashed blue curve in Fig. 6b). The continuing outbreak results in further stock-outs and an eventual OPV restart when the outbreak spreads to other blocks. Thus, an insufficiently large stockpile of readily available vaccine can under some circumstances lead to very serious consequences and a failure to maintain a world free of LPV transmission.

The last row of Table 3 shows that of the 27 iterations with mOPV stock-outs, 5 resulted in new OPV restarts that did not occur for the base case, which nearly quadruples the average expected number of cases. Moreover, remarkably the delayed oSIAs associated with stock-outs resulted in more mOPV demand and use, with a higher risk of mOPV exportations and new iVDPV introductions.

Table 4 shows the number of iterations with more than 100 or 500 million cumulative stockpile doses and the number of iterations with at least one expected mOPV stock-out for all outbreak response choices considered. Notably, extending mOPV use through T_end_ only resulted in one additional stock-out because mOPV use beyond the first 5 years remained relatively rare and successful in rapidly containing outbreaks. Using a greater geographical scope naturally increased the stockpile vaccine needs and number of stock-outs compared to the base case. More surprisingly, reducing the scope also increased the number of stock-outs. This occurred because an insufficiently large scope can result in more subsequent outbreaks in other subpopulations and blocks that then require more mOPV oSIAs. Similarly, increasing the response time moderately increased the number of stock-outs. Finally, we did not record stock-outs for the alternative oSIA vaccine options for serotype 2 outbreaks before OPV13 cessation because of the changing vaccine over time. However, for IPV oSIAs, we observed a very large number of iterations requiring at least 100 million IPV doses because of the poor ability of IPV oSIA to control the outbreaks and prevent further IPV oSIA needs.

**Table 4:** Number of iterations with vaccine needs that exceed the expected stockpile doses for different outbreak response choices

Outbreak response choice	Number of iterations that require > 100 million doses					Number of iterations that require > 500 million doses					Number of iterations with expected mOPV stockout			
	mOPV1	mOPV2	mOPV3	tOPV	IPV	mOPV1	mOPV2	mOPV3	tOPV	IPV	mOPV1	mOPV2	mOPV3	Any
Base case	23	12	8	N/A	6	1	0	1	N/A	2	19	11	7	27
Duration of mOPV use after homotypic OPV cessation (years)														
- 3	20	11	7	N/A	17	0	0	1	N/A	11	18	11	7	27
- through T_end_	24	12	9	N/A	1	0	1	1	N/A	0	19	11	8	28
Minimum R_0_ to trigger block-wide response														
- 8	32	18	14	N/A	8	2	0	1	N/A	3	28	16	13	44
- 13	20	11	11	N/A	6	2	0	1	N/A	3	17	9	11	30
Response delay (days)^a^														
- Always 30	22	13	8	N/A	5	1	0	0	N/A	2	18	11	7	26
- Always 45	23	12	8	N/A	6	1	0	1	N/A	3	19	11	7	27
- Always 60	24	12	8	N/A	8	2	0	1	N/A	5	20	11	7	28
oSIA vaccine between OPV2 and OPV13 cessation for serotype 2 outbreaks														
- tOPV	23	0	8	11	6	1	0	1	0	2	N/A	N/A	N/A	N/A
- IPV	23	12	8	N/A	21	1	0	1	N/A	3	N/A	N/A	N/A	N/A

*Abbreviations*: *IPV* inactivated poliovirus vaccine, *iVDPV* immunodeficiency-associated vaccine-derived poliovirus, *mOPV(1,2,3)* monovalent OPV (serotype 1, 2, or 3, respectively), *OPV* oral poliovirus vaccine, *OPV## cessation* globally-coordinated cessation of OPV containing the serotype(s) indicated by ##, *oSIA* outbreak response supplemental immunization activity, *R*_*0*_ basic reproduction number, *T*_*end*_ end of the analytical time horizon (i.e., December 31, 2052), *tOPV* trivalent OPV

^a^All alternative choices assume non-adaptive surveillance quality (detection threshold) and response delay in the event of a subpopulation-specific response

## Discussion

This analysis highlights the existence of a large number of outbreak response choices that influence the expected polio cases, costs, and success of long-term poliovirus risk management. OPV cessation will lead to unprecedented conditions that include increasing numbers of individuals with no recent LPV exposure and lower population immunity to transmission globally than ever existed [17]. The global model suggests that we can expect aggressive outbreak response to contain outbreaks that may occur and to protect the achievement of global polio eradication. After OPV cessation, outbreak virus exportations to other populations may much more readily lead to new outbreaks than outbreak virus exportations before OPV cessation, because of the decreasing global population immunity to transmission. Consequently, controlling outbreaks after OPV cessation requires much more aggressive response than current strategies. This includes high-quality surveillance to avoid missing signals and rapidly confirm outbreaks to minimize outbreak response delays. Delaying the response time from 45 to 60 days more than doubled the risk of uncontrolled outbreaks, which means that failure to immediately respond with population-wide vaccination to a signal while awaiting details about its origin may lead to very serious consequences. Similarly, particularly in high-risk populations, effective outbreak response requires a large geographical scope, with little expected effect of a local vaccination response given the large number of asymptomatic poliovirus infections and the ability of polioviruses to spread rapidly in the context of low population immunity to transmission after OPV cessation. In the same context, attempting to first respond with IPV may not prevent further transmission of the outbreak virus, as experienced after an initial IPV response to a WPV1 detection in Israel [18, 19], making ultimate control with OPV much more difficult and risky.

Aggressive outbreak response can in some cases reduce the stockpile vaccine requirements by preventing further spread that would require additional doses for response. The global model suggests the currently planned stockpile of 100 million filled and 400 million bulk mOPV doses of each serotype will meet the oSIA demand in over 50 % of the iterations. While mOPV stored in frozen bulk remains stable for decades, the shelf life of filled OPV products equals 2 years and at some point mOPV use after OPV cessation poses a greater risk due to its potential exportation, which we assume occurs 5 years after OPV13 cessation. Thus, several hundreds of millions of OPV doses (compared to a current annual OPV demand of billions of doses) may remain unused. However, the potentially very large human, financial, and political consequences in the event of an insufficiently large stockpile may justify some investment in a larger stockpile than likely needed, even if it means eventually wasting some OPV. Similarly, mechanisms to store larger amounts of bulk OPV or to maintain a warm base of OPV production by using them as Sabin IPV seed strains, which provide a safer option for IPV production than WPV seed strains [2, 20], may prove prudent for the eventuality of a need to restart OPV use in most developing countries in the event of a failure. Alternatively, we see real value in efforts to ensure a shorter filling delay than 1 year in an emergency situation, which would significantly reduce the probability of stock-outs.

The global model suggests a relatively small risk of exportations of mOPV used for oSIAs, even many years after OPV cessation. Different assumptions about spread between populations may increase this probability, although our results thus far suggest that doing so would disproportionately increase the risk of exporting the outbreak virus compared to mOPV viruses. The global model assumes relatively closed subpopulations that allow for selection of well-defined outbreak populations that interact relatively little with other subpopulations. Such clear-cut determination of a relatively closed target population may not prove feasible in a real outbreak situation. We do not account for the potential higher risk of exporting mOPV at the edges of the outbreak response than overall between populations, but this remains an important topic of further research that could alter our findings related to the duration of safe mOPV use. Similarly, our deterministic model for die-out of transmission may lead to die out of some effective exportations of OPV-related virus before any significant OPV evolution can occur, which represent a simplification of reality that stochastic models could address more realistically. The possibility of generating new long-term iVDPV excretors due to mOPV use appears of some concern and reinforces the current path of not using mOPV for longer than a few years after OPV cessation. Balancing the risks associated with mOPV use against the likely inability of IPV to control outbreaks in settings conducive to fecal-oral transmission [18, 19] remains an important challenge.

The results of this analysis depend on a number of previously explored and considered assumptions and limitations of the poliovirus transmission and OPV evolution model, which the global model carries forward into this analysis [2, 9, 10, 12, 21, 22]. Besides the outbreak response strategy itself, the rate of decrease in population immunity with IPV-only routine immunization and the assumed frequency of poliovirus exportations to other subpopulations and blocks determine the ability to control outbreaks and prevent mOPV from starting new VDPV outbreaks. Other limitations include the conceptual characterization of global variability using 710 subpopulations and the simplified modeling constructs used to simulate die-out, OPV evolution, waning of immunity to transmission, and effective poliovirus introduction into the deterministic poliovirus transmission and OPV evolution model [2, 12]. Moreover, the true prevalence of long-term iVDPV excretors and other long-term risks remain uncertain and effective polio antiviral drugs may offer a tool to clear long-term iVDPV infections if given to both paralytic and asymptomatic long-term iVDPV excretors [2, 7, 23], including those infected before OPV cessation or with mOPVs used for outbreak response. Other new technologies in the pipeline such as genetically stable OPV vaccines, non-replicating IPV seed strains, or IPV vaccines with enhanced ability to provide intestinal immunity may impact future risk and the options for outbreak response.

Given the uncertainty about the probability and consequences of outbreaks after OPV cessation, all interventions to prevent or minimize the probability of poliovirus reintroductions remain a priority (e.g., polio antiviral drugs, non-replicating IPV seeds strains, high bio-containment levels). Nevertheless, poliovirus reintroductions will remain possible and this analysis clearly demonstrates the importance of outbreak response and stockpile preparedness to maximize the probability of maintaining a polio-free world after OPV cessation.

## Conclusions

Numerous outbreak response choices affect the probability of successfully managing poliovirus risks immediately after OPV cessation and in the long term. Speed and quality of oSIAs unambiguously improve the ability to manage risks, and stockpiling more vaccine than expected for outbreak response appears a prudent approach. While OPV appears the right choice to respond to outbreaks with a few years of OPV cessation, longer-term vaccine type decisions remains challenging since OPV comes with VDPV risks while IPV comes with high costs and no demonstrated ability to control outbreaks in settings conducive to fecal-oral poliovirus transmission. Outbreak response strategies and vaccine choices will remain critical in the polio endgame.

## Abbreviations

cVDPV(1,2,3): circulating VDPV (serotype 1, 2, or 3, respectively)
DEB model: differential equation-based poliovirus transmission and OPV evolution model
EIPM: mixing-adjusted effective immune proportion
GPEI: global polio eradication initiative
IPV: inactivated poliovirus vaccine
iVDPV(1,2,3): immunodeficiency-associated VDPV (serotype 1, 2, or 3, respectively)
LPV: live poliovirus
mOPV(1,2,3): monovalent OPV (serotype 1, 2, or 3, respectively)
OPV: oral poliovirus vaccine
OPV## cessation: globally-coordinated cessation of OPV containing the serotype(s) indicated by ##
oSIA: outbreak response SIA
PID: primary immunodeficiency disease
R_0_: basic reproduction number
RI: routine immunization
R_n_: net reproduction number
SIA: supplemental immunization activity
T_end_: end of the analytical time horizon (i.e., December 31, 2052)
tOPV: trivalent OPV
VDPV: vaccine-derived poliovirus
WPV(1,2,3): wild poliovirus (serotype 1, 2, or 3, respectively)
